# Surgical outcomes of the closure of mesenteric defects in side-to-side jejunoileal anastomosis plus proximal loop ligation (SSJIBL) using absorbable and non-absorbable surgical sutures

**DOI:** 10.3389/fsurg.2025.1650828

**Published:** 2025-09-16

**Authors:** Yonglin Li, Jing Wang, Chengyu Wu, Xiaojing Lu, Qi Zheng, Rongwei Wei, Ziliang Zong, Yigang Chen

**Affiliations:** ^1^ Department of General Surgery, Wuxi No. 2 Hospital, Jiangnan University Medical Center, Wuxi, China; ^2^ Department of General Surgery, The Affiliated Wuxi No. 2 Hospital of Nanjing Medical University, Wuxi, China

**Keywords:** SSJIBL, mesenteric defect, mesenteric hiatal hernia, absorbable suture, non-absorbable suture

## Abstract

**Background:**

Mesenteric hiatal hernia represents a significant complication following gastrointestinal surgery, and the closure of mesenteric defects has been shown to mitigate the risk of such hernias. SSJIBL, a surgical technique that has gained prominence in recent years, is widely acknowledged for its efficacy in glucose reduction and its association with fewer complications. Nevertheless, there remains a gap in the literature regarding the optimal suture choice for closing mesenteric defects, as no definitive studies or reports have addressed this specific issue to date. So we wanted to know what sutures we could use to more safely close the mesenteric defect.

**Materials and methods:**

36 New Zealand rabbits were divided into three groups, NC, Absorbable suture and Non-absorbable suture. Group NC was not operated, group Absorbable suture close the mesenteric defect with absorbable suture, and group Non-absorbable suture close the mesenteric defect with non-absorbable suture; the rabbits were weighed and measured monthly, and after three months, the rabbits were observed to see if there was any internal hernia and to detect the tethered lacunae tension.

**Results:**

According to the results of the experiment, both absorbable suture and non-absorbable suture can induce infiltration of inflammatory cells and enhance adhesion strength relative to the NC group.

**Conclusion:**

Both absorbable and non-absorbable sutures are safe and reliable.

## Introduction

Mesenteric hiatal hernia is a significant complication that can arise following gastrointestinal surgery. Its occurrence often leads to severe clinical consequences, necessitating additional surgical intervention (1). Research has demonstrated that the closure of mesenteric defects after gastrointestinal surgery effectively reduces the likelihood of developing mesenteric hiatal hernia (2, 3).

Side-to-side jejunoileal anastomosis plus proximal loop ligation (SSJIBL) has replaced jejunoileal bypass (JIB) as a new type of weight loss surgery option for the treatment of severe obesity, which was confirmed that can induced better glucose-lowering effects than the other bariatric surgical procedure (4, 5).

Absorbable suture has been shown effectively and safely to close the mesenteric defect in gastric bypass surgery (6). However, to date, limited research has been conducted to determine the most suitable surgical suture for closing the mesenteric hiatus in SSJIBL. To address this gap, we will perform SSJIBL on New Zealand rabbits and close the mesenteric defect using two distinct types of surgical sutures: absorbable surgical barbed suture and non-absorbable surgical suture. This study aims to evaluate the impact of each suture type on the postoperative closure efficacy and the tensile strength at the site of membrane adhesion.

In this study, we established SSJIBL model in New Zealand rabbits and used different sutures to suture the mesenteric defect to investigate whether using non absorbable sutures and absorbable sutures to close the mesenteric defect can produce safe adhesion.

## Materials and methods

This study was approved by the ethical committee of the Jiangnan University Affiliated Central Hospital. All applicable institutional and national guidelines of the People's Republic of China for the care and use of animals were followed.

### Animal model

A total of 36 male New Zealand White Rabbit weighting 2.77 ± 0.15 kg were purchased from New Zealand. All rabbits were maintained separately in galvanized wire pens (35 × 35 × 60 cm) with freely accessible feeders (*ad libitum*) and a freshwater outlet under the same management and hygienic conditions, namely a regimen of 12 h light and 12 h dark, natural ventilation, an average temperature of 25°C and a relative humidity of 50%. All surgeries were performed under chloral hydrate anesthesia, and all efforts were made to minimize suffering.

### Suture material

4-0 non-absorbable suture (Prolene Polypropylene Suture) and 4-0 absorbable suture (STRATAFIX™ Symmetric Monocryl™ Plus) from Ethicon.

### Experimental protocol

Rabbits were randomly divided into 3 groups (*N* = 36) after a 1-week acclimation. Rabbits in all groups underwent side-to-side jejunoileal anastomosis plus proximal loop ligation except for the control group (the mesenteric defects is closed with different sutures). Body weight and food intake after surgical procedures were monitored and recorded.

### Surgical procedure

After overnight fasting and water deprivation, the animals were anesthetized with pentobarbital sodium intravenously at the ear margin (30 mg/kg). The abdomen was shaved and an incision of approximately 10 cm was made from the subxiphoid process to the mid-abdomen, thereby entering the abdominal cavity.

### Side-to-side jejunoileal bypass plus proximal loop ligation

**Figure 1:**
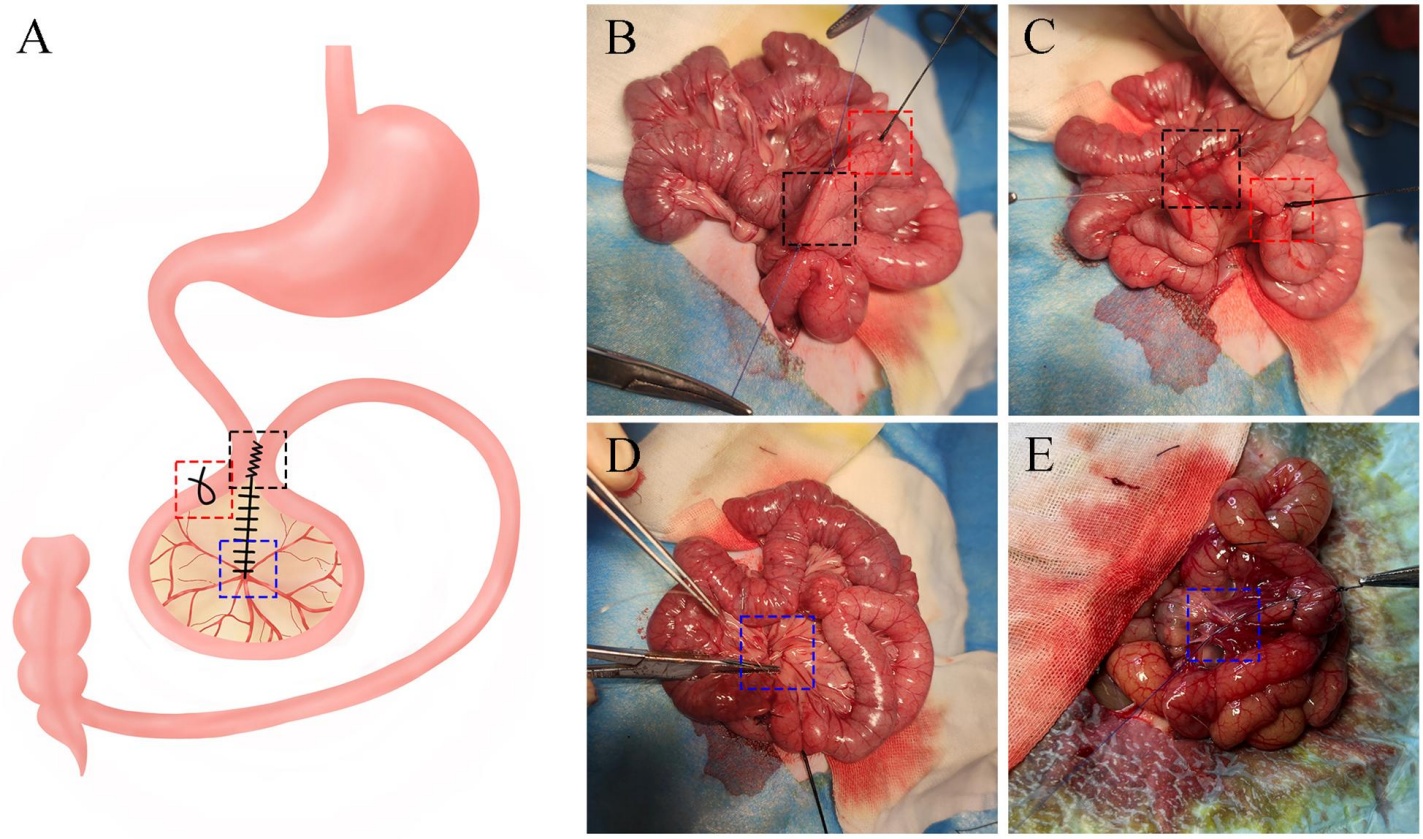
Illustration of SSJIBL **(A)** black, red, and blue boxes represent anastomosis, ligatures, and mesenteric defects that were closed (B&C) isoperistaltic side-to-side anastomosis and ligate the intestinal, using 4-0 absorbable suture **(D)** and 4-0 non-absorbable suture **(E)** to close the mesenteric defect.

The total length of the small intestine, measured from the Treitz ligament to the ileocecal valve, was recorded, and 30% of this length was calculated. Starting 15 cm distal to the Treitz ligament, an isoperistaltic side-to-side anastomosis was performed with the distal small intestine to restore intestinal continuity (bypassing 30% of the total small bowel length). The enteric anastomoses were completed using 3-0 absorbable sutures. Subsequently, the intestinal tract was ligated 1 cm away from the anastomotic site with 0 silk suture (Figures 1A–C). The mesenteric defect was then closed using continuous suturing, with 4-0 non-absorbable suture in one group (Figure 1D) and 4-0 absorbable suture in another (Figure 1E). Finally, the abdominal cavity was closed in two layers using 3-0 non-absorbable sutures.

### Sham operations

Following skin preparation and disinfection, a 10-cm midline incision was made to access the peritoneal cavity, and the intestine was gently manipulated. The abdominal cavity was closed in two layers using 3-0 surgical sutures.

The operative time for rabbits in all groups was maintained within 12 ± 10 min. Postoperatively, the animals were placed in individual cages to recover from anesthesia. All rabbits were fasted for the first 24 h following surgery, after which they were provided unrestricted access to standard chow and tap water from the first postoperative day until the conclusion of the experiment.

All rabbits were weighed once a month and euthanized by exsanguination under deeply anesthetize (30 mg/kg pentobarbital sodium intravenously at the ear margin) after 3 months. The lifespan of a New Zealand rabbit is approximately 7–8 years, compared to about 80 years in humans (7). A 3-month experimental period in rabbits is roughly equivalent to 2.5–3 years in humans. Therefore, we consider the 3-month observation period to be justified.

The abdominal cavity was opened to examine the mesenteric defect adhesions and assess any changes in the suture material in both groups. The degree of adhesion was evaluated using a semi-quantitative grading method based on Blauer's criteria (8) (Table 1).

**Table 1:** Blauer's semi-quantitative grading method.

Grade	Description
0	No adhesions
1	Thin or narrow, easily separable adhesions
2	Thick adhesions limited to one area
3	Thick and widespread adhesions
4	Thick and widespread adhesions, plus adhesions of viscera to anterior and/or posterior abdominal wall

**Figure 2:**
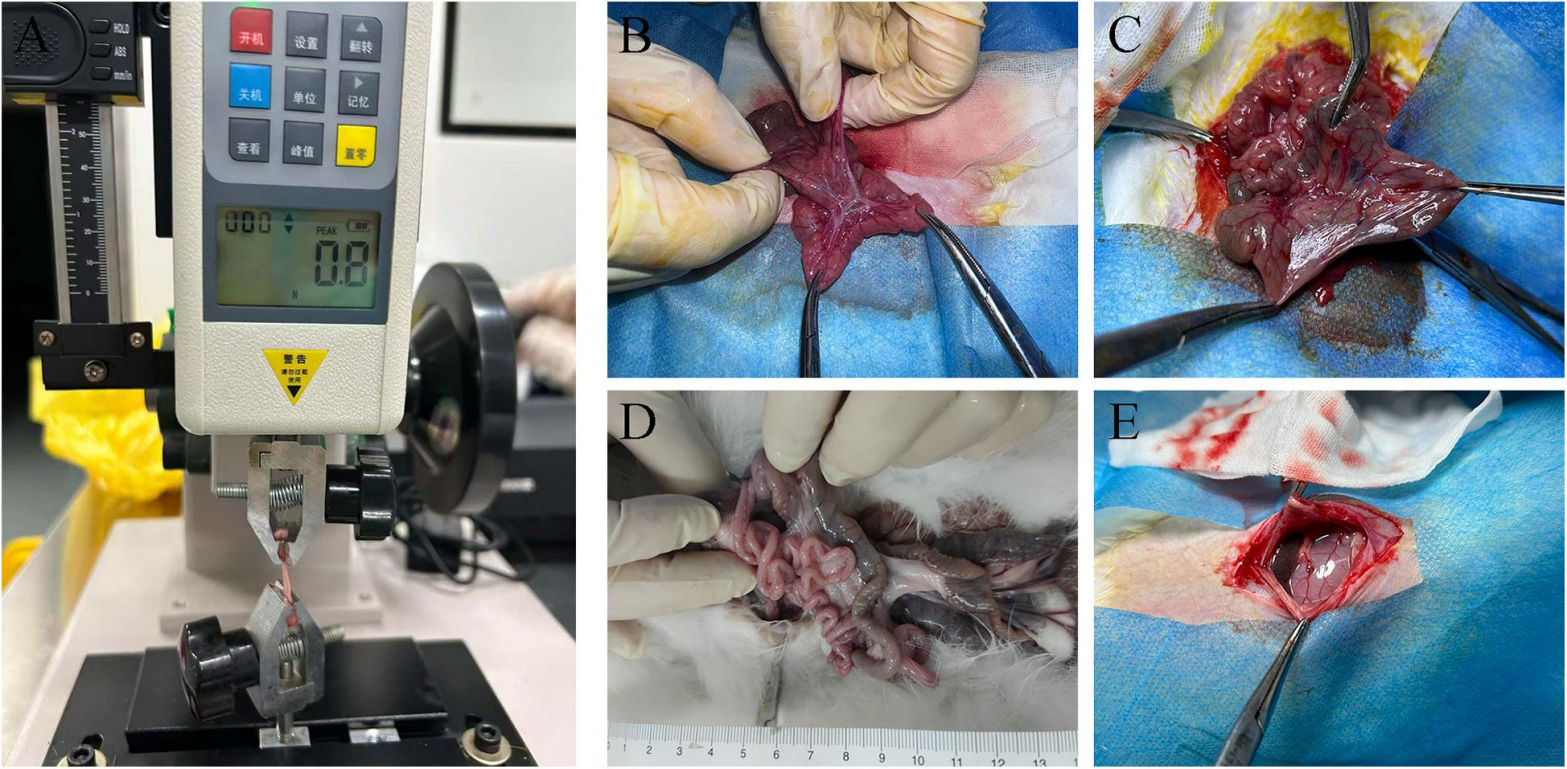
Specimen processing and surgical results **(A)** tensile tester **(B)** group absorbable suture **(C)** group non-absorbable suture **(D)** atrophied small intestine **(E)** severe ascites of one rabbit in group absorbable suture.

A 2 cm × 0.5 cm piece of mesenteric tissue at the site of normal tissue and mesenteric hiatus was taken and its maximum tension was measured with instruments for measuring tensile force (Figure 2A).

### Techniques for histopathological investigation

Mesenteric tissue samples were first fixed with a 4% paraformaldehyde solution. Tissues were obtained after fixation and paraffin blocks were obtained after routine procedures. The resulting 5 µm serial sections were stained with H&E. The sections were then viewed with an Olympus BX 51 light microscope.

### Statistics

The changes in Food intake, Body weight, and Adhesion scores are expressed as mean ± standard deviation (SD).Differences between the groups were assessed by one-way analysis of variance (ANOVA, LSD post-test). *P* < 0.05 was assumed significant difference. Statistics were performed using SPSS, version 18.0, statistical software (SPSS Inc., Chicago).

## Results

### Operative results

The SSJIBL model was successfully established in all rabbits within the experimental groups. All animals survived the surgical procedure and completed the 12-week follow-up period (Figures 2B,C). Notably, we observed that the bypassed segments of the small intestine exhibited varying degrees of atrophy in nearly all groups after 12 weeks (Figure 2D). Additionally, in Group Absorbable Suture, one rabbit developed severe ascites following the SSJIBL procedure (Figure 2E).

### IH and adhesion of the mesenteric defects

All New Zealand rabbits were dissected after 12 weeks, and no internal hernia was observed in both experimental and negative control group.

**Figure 3:**
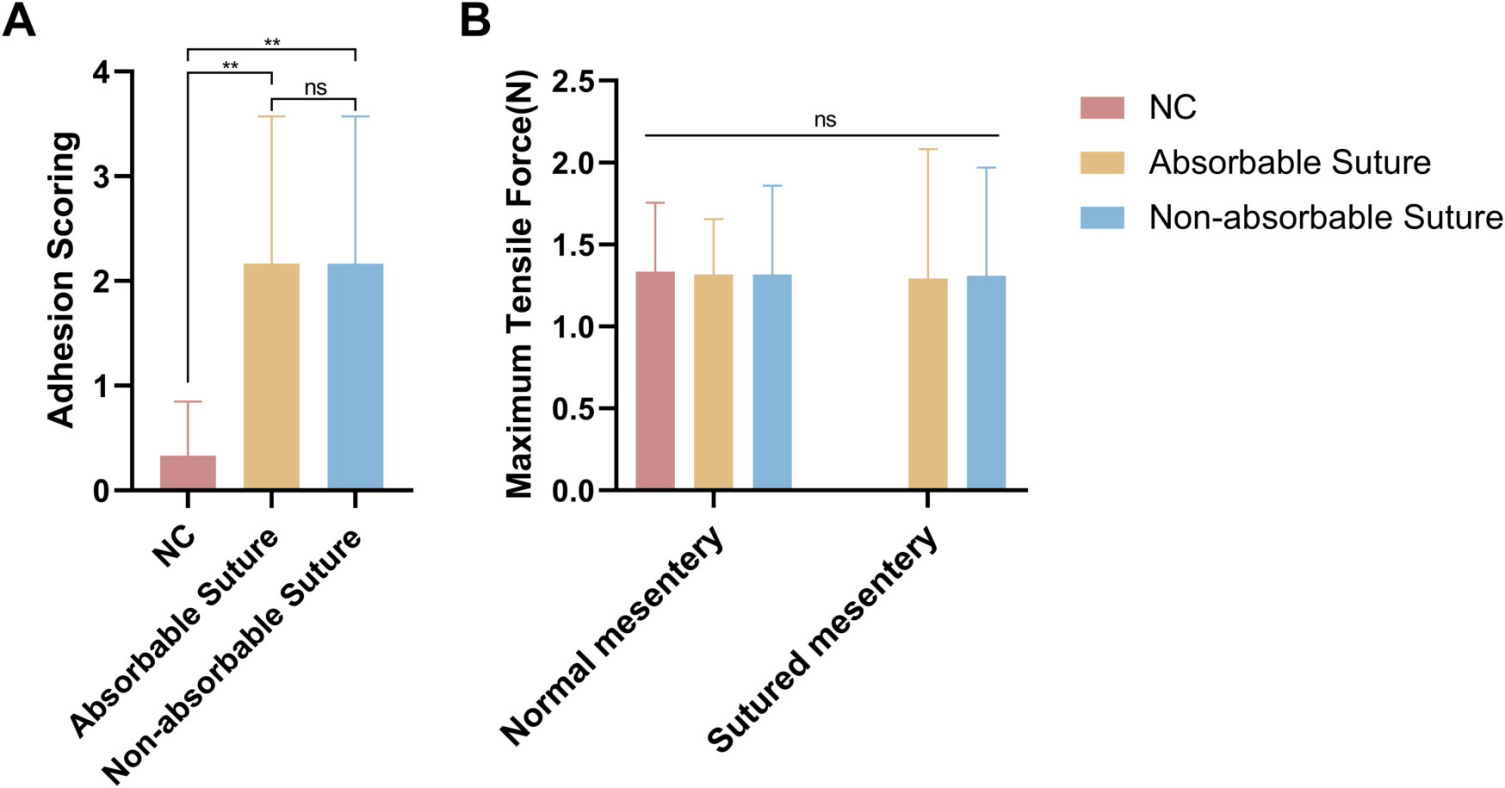
Histogram of surgical results **(A)** adhesion scores and **(B)** maximum tensile force for the 3 groups.

Adhesion scores were significantly higher in groups Absorbable Suture and Non-absorbable Suture than in group NC, but there was no significant difference between groups Absorbable Suture and Non-absorbable Suture (Figure 3A).

The maximum maximal tension of the normal mesenteric tissue was similar to that of the mesenteric tissue at the site of the closed mesenteric hiatal hernia and was not statistically different, both in group Absorbable Suture and group Non-absorbable Suture (Figure 3B).

### Histopathological investigation

**Figure 4:**
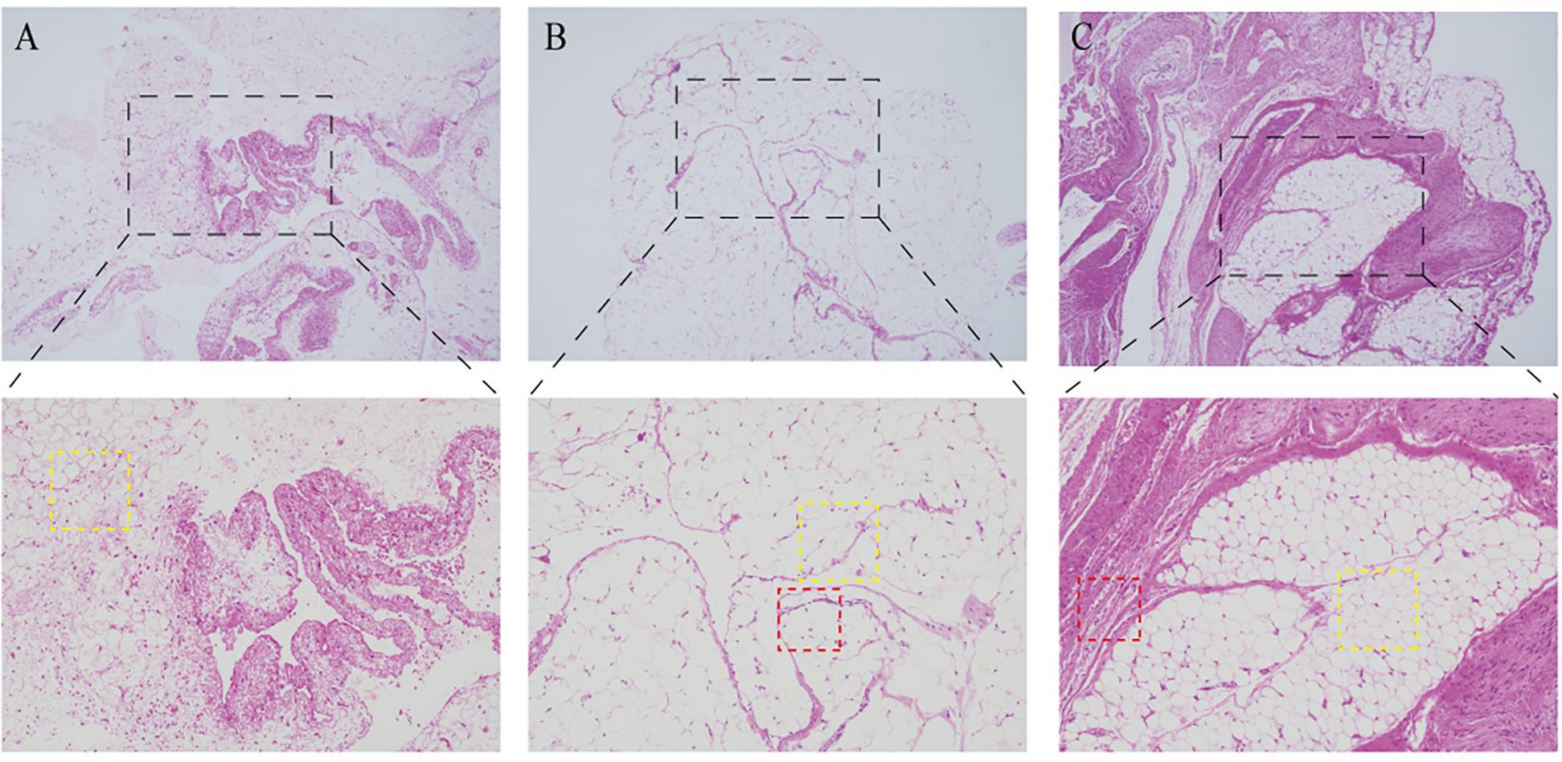
H&E staining **(A)** group NC **(B)** group absorbable suture **(C)** group non-absorbable suture (yellow and red boxes represent adipocytes and inflammatory cells).

Based on the H&E staining, we can see adipocytes between the tissues of all three groups and an infiltration of inflammatory cells in both Group Absorbable Suture and Non-absorbable Suture, which, according to our guess, is the reason for the formation of firmer adhesions (Figure 4).

## Discussion

Internal hernias can occur in any surgery that alters the structure of the gastrointestinal tract. And, it has been shown that J-J defects are more likely to occur than Petersen's defects in a procedure like LRYGB without closure of the mesenteric hiatus, which reinforces the value of our study (9).

In this study, closure of the mesenteric hiatus with absorbable and non-absorbable sutures produced similar adhesion scores, both of which were significantly higher than those in the group without closure of the mesenteric hiatus, and the normal as well as the maximum tensile force of the mesentery that was sutured was similar in all three groups.

This on the one hand shows that suturing the mesenteric hiatus produces higher adhesion scores and reduces the likelihood of mesenteric hiatal hernia, and on the other hand it shows that the use of absorbable and non-absorbable sutures did not affect the adhesion scores more, but from an economic point of view, absorbable surgical sutures are more expensive than non-absorbable surgical sutures, and the use of non-absorbable surgical sutures can reduce the financial burden on the patient.

However, we need to emphasize that producing higher adhesion scores can also have drawbacks, and there is evidence that severe intestinal adhesions may cause small bowel obstruction, which also needs to be weighed by the surgeon, but in a sense, acute intestinal obstruction caused by a mesenteric hiatal hernia may be more of a concern than chronic intestinal obstruction that resolves with conservative management.

In addition, in group Absorbable sutures, one rabbit developed severe ascites. Ascites is divided into portal hypertensive ascites and non-portal hypertensive ascites (10). We hypothesize that there are two possibilities for the production of ascites, one of which is transudate due to obstruction of venous return caused by closure of the peritoneal lacunae, and the other is inflammatory ascites due to inflammation around the sutures. However, the operator did not retain a specimen of ascites, so we were unable to assay this and determine its nature.

According to studies, the pathophysiology of adhesion formation seems to be related to a local inflammatory response (11), and in histopathological testing of the mesentery, we found infiltration of inflammatory cells in both groups but not in the control group, suggesting that both absorbable sutures and non-absorbable sutures can cause infiltration of inflammatory cells and produce reliable adhesions.

## Conclusion

Closure of mesenteric hiatal hernias with absorbable and non-absorbable sutures produces similar adhesion scores, and the maximum tension of the mesentery at the point of closure of the mesenteric hiatus is similar to that of the normal mesentery, and surgeons choosing either absorbable or non-absorbable sutures for closure of the mesenteric hiatus increase the inter-intestinal adhesion and reduce the incidence of mesenteric hiatal hernias; however, choosing a non-absorbable suture is more likely to reduce the economic burden on the patient.

## Data Availability

The original contributions presented in the study are included in the article/Supplementary Material, further inquiries can be directed to the corresponding author.
