# Supplementary figures and images for: Surgical outcomes of the closure of mesenteric defects in side-to-side jejunoileal anastomosis plus proximal loop ligation (SSJIBL) using absorbable and non-absorbable surgical sutures
Source: Front Surg. 2025 Sep 16;12:1650828. doi: 10.3389/fsurg.2025.1650828 (PMC12479435; doi:10.3389/fsurg.2025.1650828)

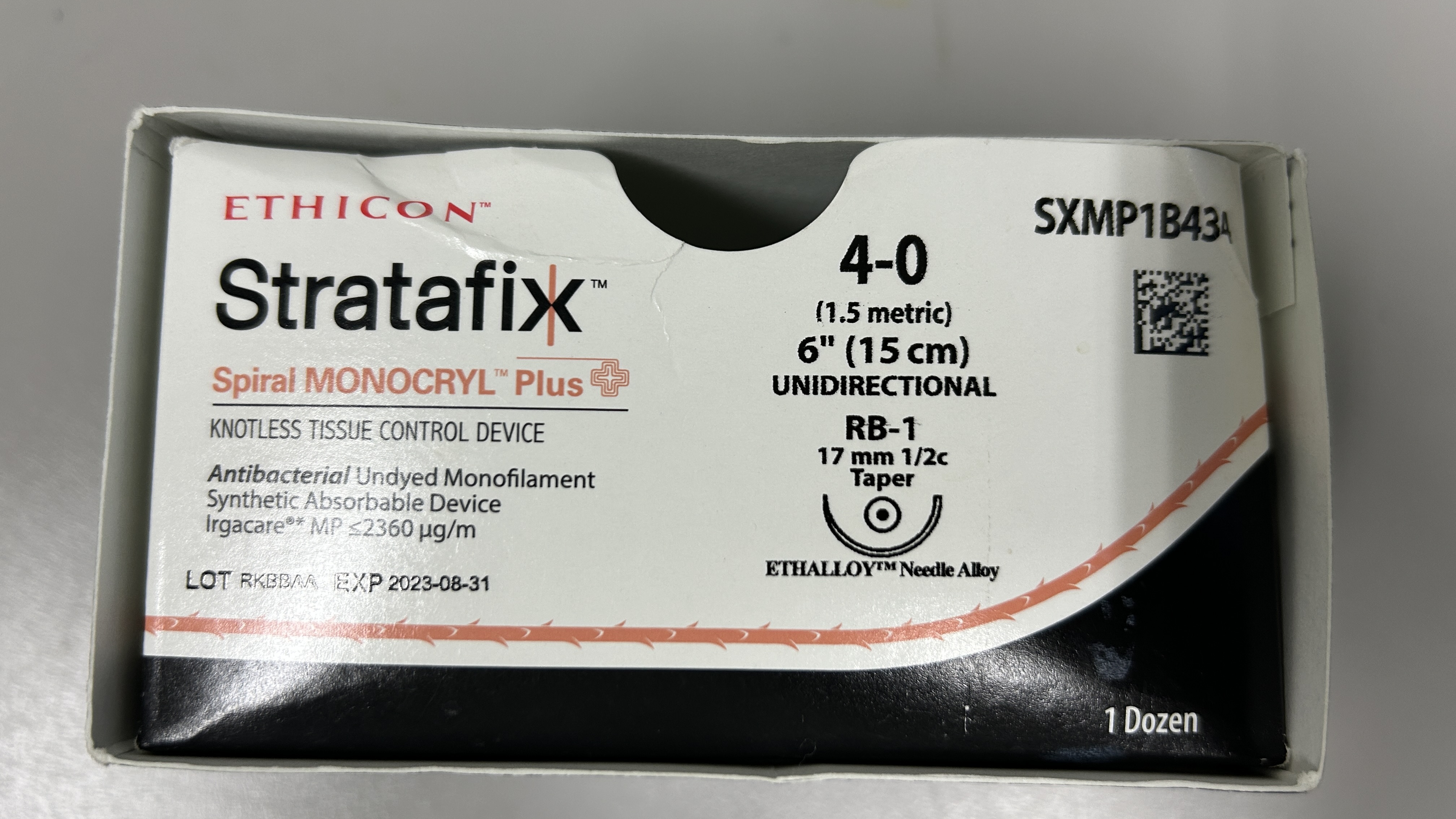

Supplement: Supplementary file 1 [file Image1.jpeg]

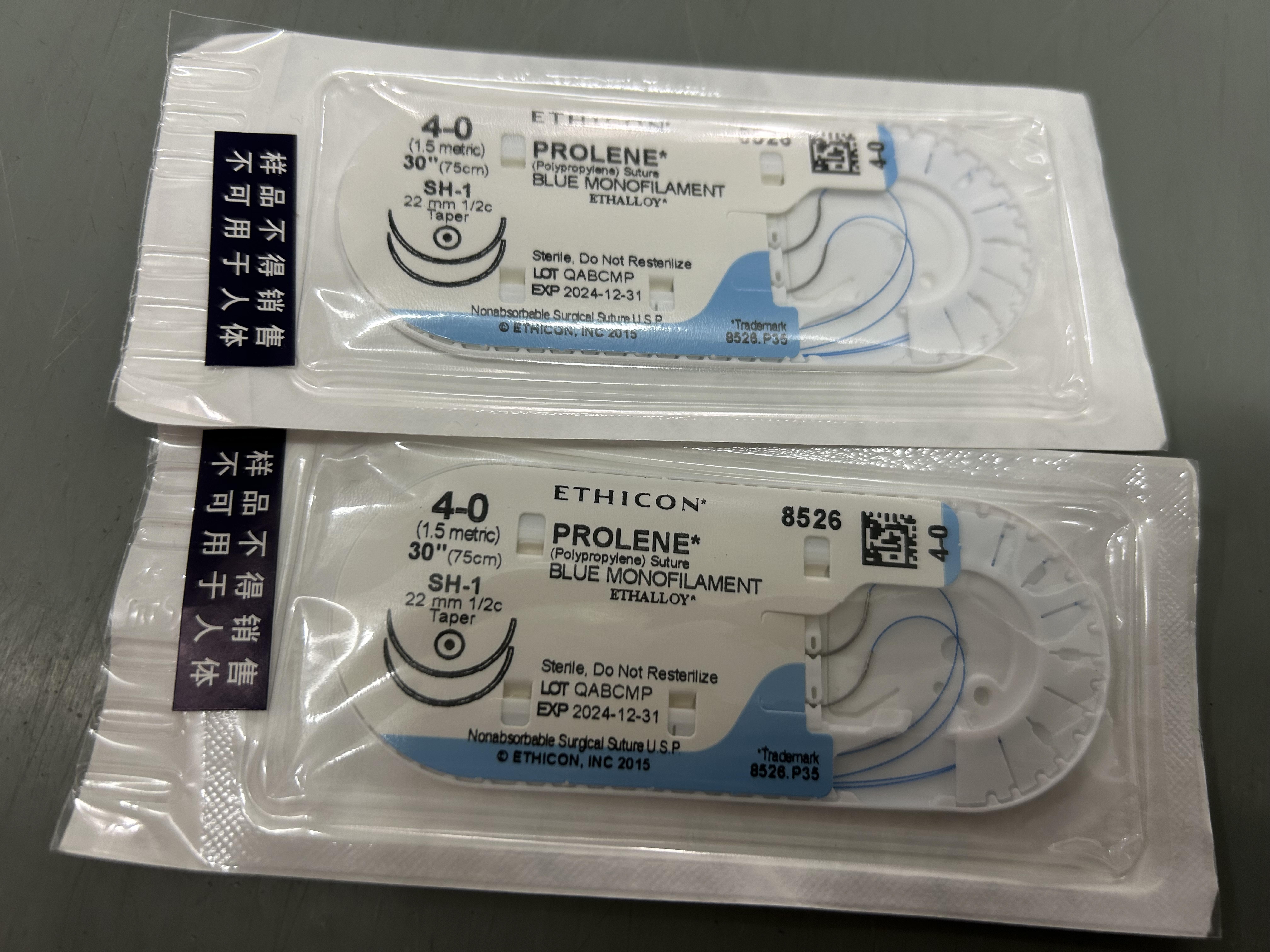

Supplement: Supplementary file 2 [file Image2.jpeg]
